# Diagnosis of a Large Vaginal Cyst During Pregnancy

**DOI:** 10.7759/cureus.78402

**Published:** 2025-02-03

**Authors:** Duaa M Al Abbas, Fatema J Shaikh Majed, Shahd A Alramadhan

**Affiliations:** 1 Obstetrics and Gynaecology, Eastern Health Cluster, Minstry of Health, Qatif, SAU; 2 Obstetrics and Gynaecology, Salmaniya Medical Complex, Manama, BHR; 3 Medicine, Faculty of Medicine, Royal College of Surgeons in Ireland, Dublin, IRL

**Keywords:** case report, mullerian cyst, obstetrics complication, threatened preterm labor, vaginal cyst, vaginal mass in pregnancy

## Abstract

In this report, we detail an unusual presentation of a large Müllerian cyst during pregnancy. A primigravida woman, 25 weeks and 6 days pregnant, arrived at the Obstetrics and Gynecology Emergency Department at Qatif Central Hospital, Saudi Arabia, complaining of something protruding from her vagina, accompanied by a yellowish vaginal discharge and mild back pain. After a series of initial examinations and tests, the patient was diagnosed with cervical incompetence and prolapsed fetal membranes, indicating a threat of preterm labour. However, after a lengthy hospital stay, multiple readmissions, and inconsistencies in the patient’s history that contradicted the initial diagnosis, there was a rising suspicion about the nature of the vaginal mass. The patient was re-evaluated with a detailed examination and targeted pelvic ultrasound, which led to the diagnosis of a vaginal cyst. Cyst aspiration was performed before labour, but the patient was unable to deliver vaginally. Consequently, she underwent an emergency cesarean section, followed by an excision of the vaginal cyst. Histopathological analysis finally revealed that the cyst was a Müllerian cyst.

## Introduction

Vaginal cysts are estimated to have a prevalence of less than 1%. These can be classified as either congenital or acquired. Müllerian cysts, the most common type of congenital cyst of embryological origin, account for up to 40% of cystic masses [1,2]. Indeed, their exact prevalence is likely underestimated due to their often asymptomatic nature [3]. Most Müllerian cysts are small in size (typically < 2 cm) and are found incidentally during pelvic examinations, imaging, or delivery [4]. However, in rare cases, Müllerian cysts can exceed 4 cm and cause discomfort, a sensation of heaviness in the perineal region, vulva swelling, vaginal discharge, and difficulties with voiding or defecation [2,5]. They tend to occur most commonly in child-bearing age [4]. During pregnancy, large, tense cysts can be misdiagnosed as protruding fetal membranes. This leads to guidance errors and prolonged patient management [6].

We detail an uncommon case involving a female in the late second trimester of pregnancy, who presented with a large Müllerian cyst mistakenly identified as a protruding fetal membrane. Initial examinations hinted at cervical incompetence, which raised suspicions of threatened preterm labour, thus obscuring the actual diagnosis.

## Case presentation

A 27-year-old female patient, primigravida, presented at the Obstetrics and Gynecology Emergency Department at Qatif Central Hospital, Saudi Arabia, 25 weeks and 6 days into her pregnancy. She voiced concerns about something protruding from her vagina. This was accompanied by a yellowish vaginal discharge and slight back pain. She negated the occurrence of abdominal pain, vaginal bleeding, fluid leakage, and urinary and gastrointestinal disruptions. In the initial examination, the patient was found to be vitally stable. She possessed a gravid abdomen that was soft and relaxed, consistent with a 26-week gestation period, and no contractions were discernible. Her detailed history revealed that she had been married for five years as her husband had been diagnosed with asthenozoospermia and had two failed in vitro fertilization trials. This was her first spontaneous pregnancy. The patient also had a history of a ruptured right ovarian cyst, which had been treated with laparoscopic cystectomy 10 years prior. Moreover, she was a known case of sickle cell disease and she was not taking hydroxyurea, with no history of vaso-occlusive crisis.

Due to the risk of rupturing a possible fetal membrane sac, a vaginal examination was intentionally foregone. A vaginal inspection revealed a smooth, dry surface, pink-coloured sac that was tense and protruding out of the vaginal opening, tentatively identified as protruding fetal membranes. The patient was admitted to the labour and delivery room as a suspected case of preterm labour. An abdominal ultrasound conducted by the on-call consultant registered a single viable fetus, measurements aligning with the gestational age, adequate amniotic fluid, a fundo-anterior placenta, and a dilated internal cervical os measuring 4 cm. The patient was initiated on magnesium sulfate for neuroprotection, supplemented with dexamethasone and ampicillin, and kept under observation.

One day after admission, the patient began to complain of difficulty passing stool, despite wanting to do so for the past three days, and reported pressure in the perineal area. A repeated local inspection of the vagina, aided by separating the labia, revealed a protruding sac almost at the +3 station. A repeated abdominal ultrasound showed a viable fetus, a cervical os 2 cm dilated, a cervical length of 1 cm, and funnelling at the cervix. Therefore, the patient was diagnosed as a late-presenting case of cervical incompetence, rendering her ineligible for emergency cervical cerclage. She remained under observation for 12 days before deciding to discharge herself against medical advice, despite understanding the associated risk.

The patient was readmitted at 34 weeks and 1 day into her pregnancy, for the first time, as a case of sickle cell disease with a vaso-occlusive crisis. She was consequently managed and reexamined through vaginal inspection, during which no sac was observed. She was then discharged in a stable condition.

At 35 weeks and 1 day into her pregnancy, the patient was admitted for the third time due to complaints of vaginal spotting. She was in a stable condition. Upon vaginal inspection, no bleeding was observed, but a sac was noticed to be protruding.

**Figure 1:**
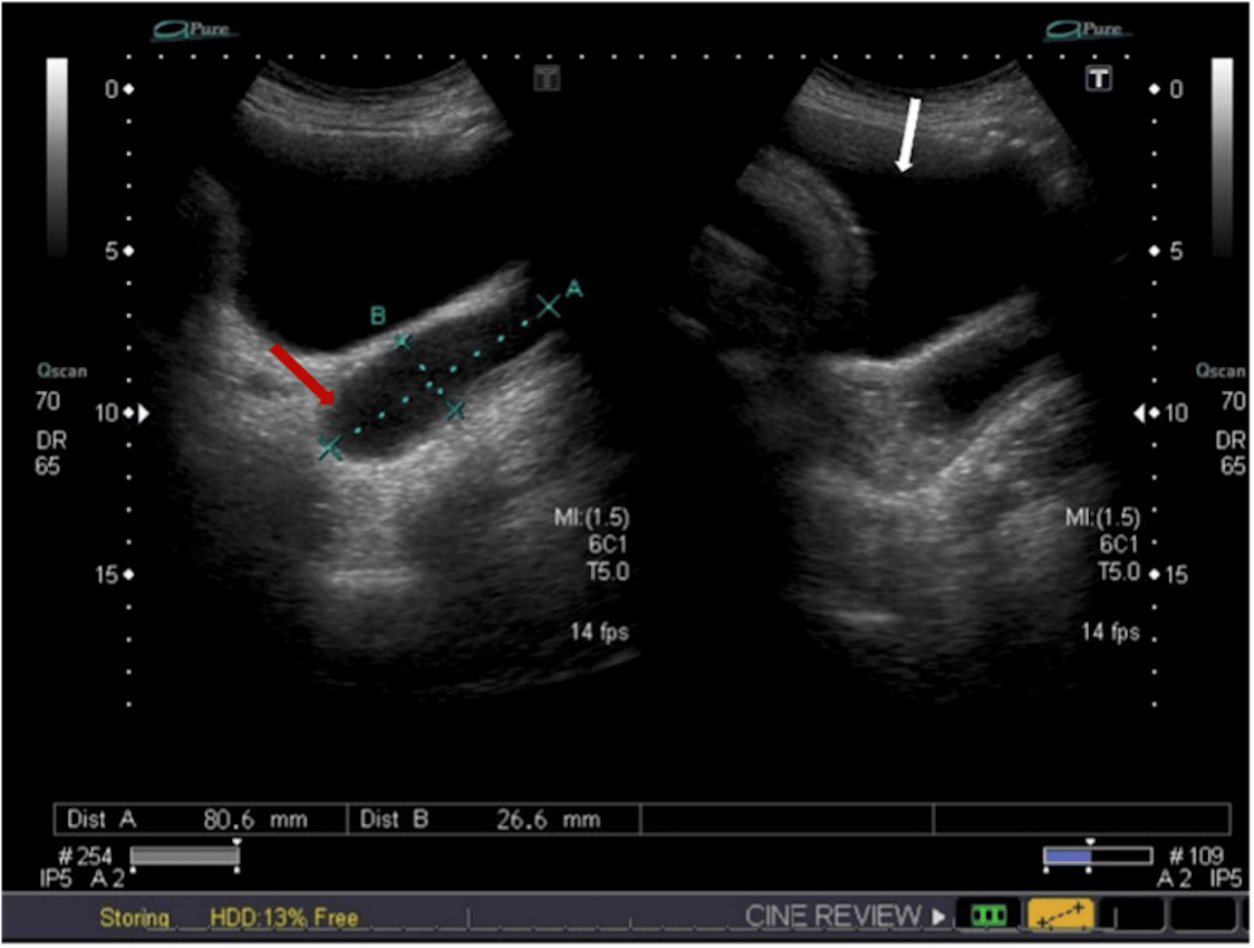
Targeted pelvic ultrasonography showing a cyst (red arrow) located in the upper vagina and extending to the cervix, with a measurement of approximately 8 cm in length, indicated by the blue line (A). The white arrow points to the urinary bladder.

**Figure 2:**
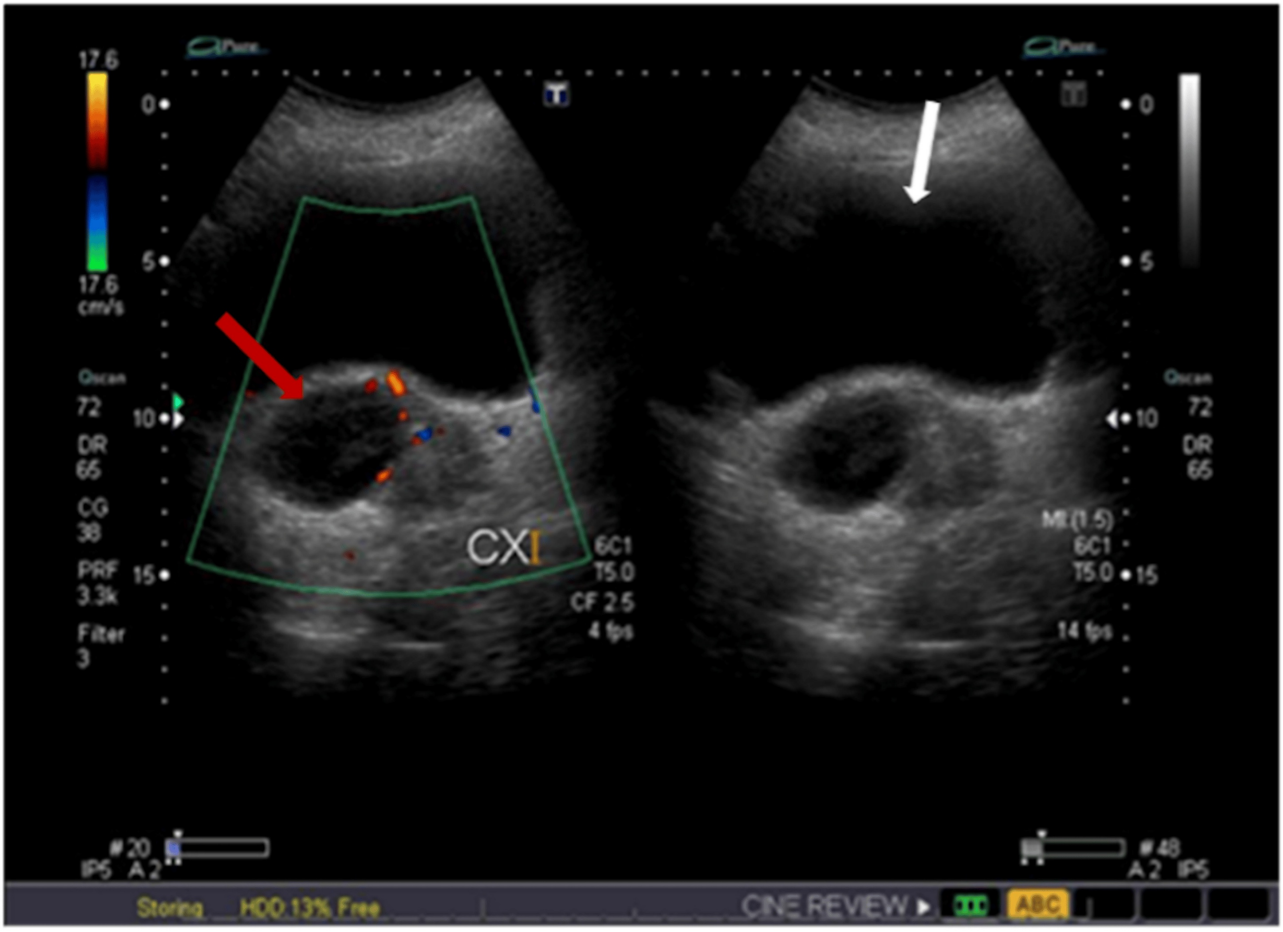
Targeted pelvic ultrasonography showing a vaginal cyst (red arrow) containing clear fluid, with no signs suggesting solid components or vascularity on Doppler imaging. The white arrow indicates the urinary bladder.

**Figure 3:**
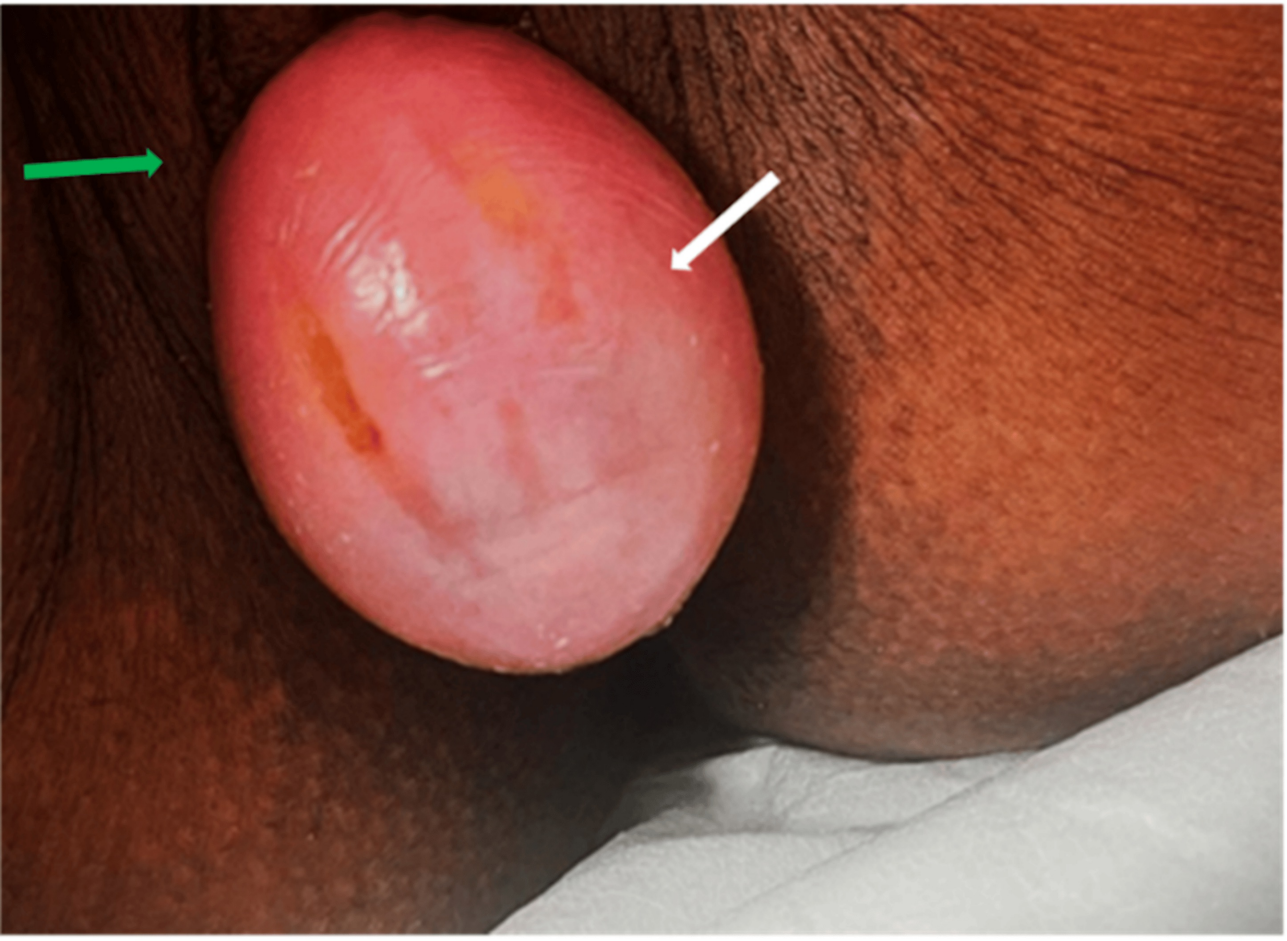
Vaginal inspection shows a pink, smooth-surfaced, tense cyst (white arrow) protruding from the vaginal opining. The green arrow points to the labia majora.

The patient’s ambiguous and extended presentation did not correspond to either protruding fetal membranes or threatened preterm labour. This discrepancy led to a deeper suspicion about the nature of the vaginal mass, prompting the medical team to re-evaluate the patient thoroughly. To explore further, a targeted pelvic ultrasound was conducted to identify the sac’s origin. The ultrasound revealed a cyst containing internal mobile fluid in the upper vagina, extending up to the cervix, and measuring 4.7 × 4.3 × 8 cm. No solid component or vascularity was apparent within the cyst (Figures 1, 2). An experienced consultant carried out a vaginal examination, disclosing a dry, smooth-surfaced reducible vaginal cyst emerging from the anterolateral wall of the vagina. The cyst was roughly 4 × 4 cm in size, with the pedicel measuring 1 × 2 cm (Figure 3). Unfortunately, ultrasound images for the initial diagnosis were not available. However, ultrasound images for the final diagnosis, which were included in our report, clearly show the large vaginal cyst. Consequently, a diagnosis of a vaginal cyst was confirmed.

**Figure 4:**
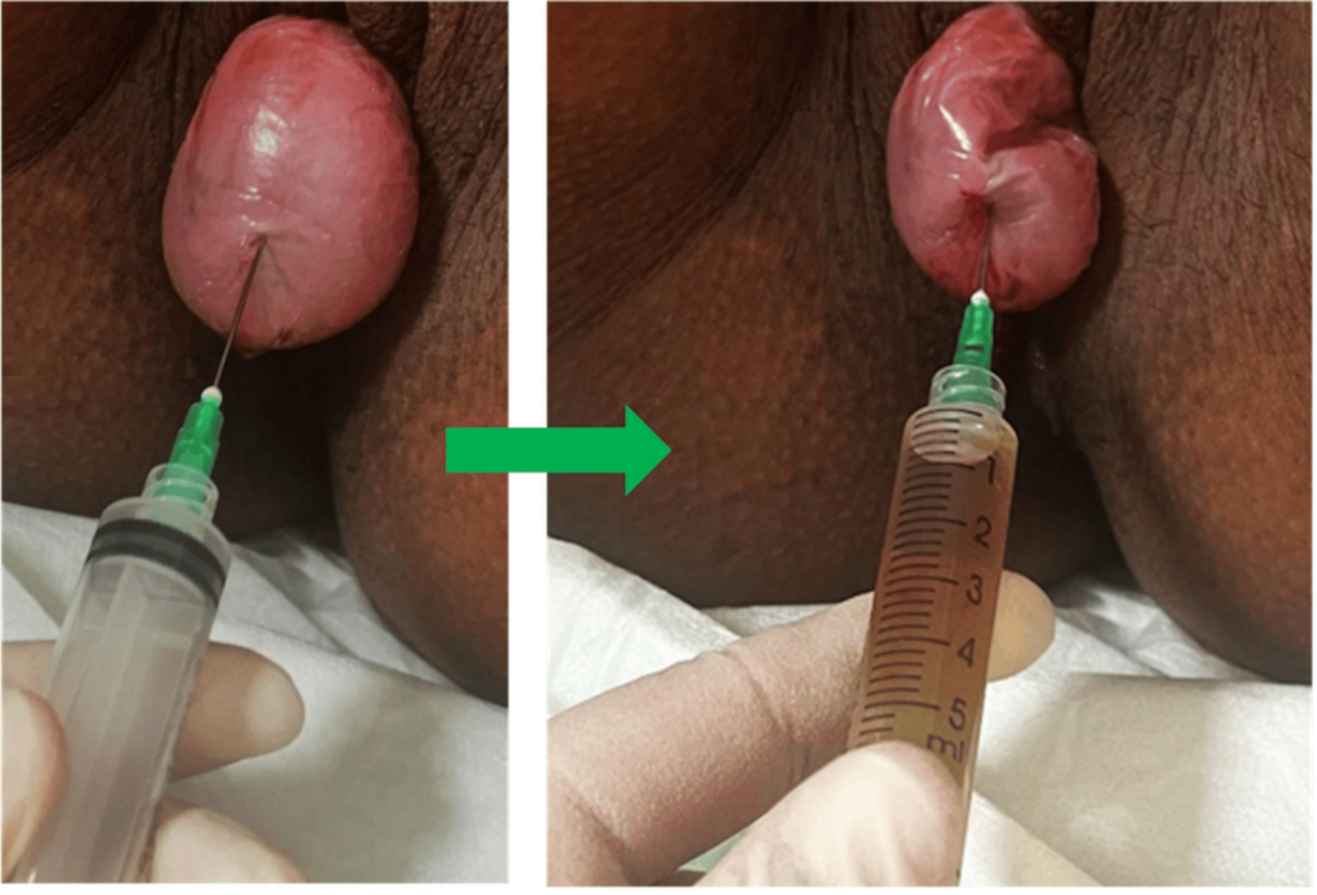
Vaginal cyst aspiration procedure performed prior to the anticipated vaginal delivery. Approximately 5 cc of yellowish fluid was drained from the cyst.

 Vaginal cyst aspiration procedure performed prior to the anticipated Vaginal delivery. Approximately 5 cc of yellowish fluid was drained from the cyst (figure 4).

At 36 weeks and 1 day of pregnancy, the patient presented to the Obstetrics and Gynecology Emergency Department with a second episode of severe sickle cell disease vaso-occlusive crisis, requiring admission to the ICU and infusion of fentanyl. The patient was subsequently managed and stabilized. During the same admission, the patient started experiencing labour pains after five days, and the cardiotocography (CTG) indicated regular contractions. A vaginal examination corroborated cervical changes, signifying the onset of labour. The patient opted for epidural analgesia. As labour advanced, an episiotomy was conducted to aid in the delivery. However, due to inadequate maternal effort, several attempts at vacuum extraction were unsuccessful. Eventually, non-reassuring features observed on the CTG necessitated an emergency cesarean section.

After delivery, the collapsed vaginal cyst sac was excised, followed by prompt repair of the vaginal wall. The excised tissue was sent for histopathological analysis. Ultimately, it was identified as a cyst primarily lined by mucinous endocervical-type epithelium, with focal tubal and squamous metaplasia, consistent with a Müllerian cyst.

## Discussion

According to the literature, this is one of the rarest documented cases of a large Müllerian cyst that presented during pregnancy and was misdiagnosed as protruding fetal membranes. Despite its rareness, this case underscores the importance of maintaining a high degree of clinical suspicion for similar presentations to avoid misdiagnosis. This misidentification could result in undue worry, unnecessary extended hospital stays, and a waste of effort and resources.

Previous research suggests that the most common differential diagnoses for vaginal cysts are Müllerian cysts (44%), epidermal inclusion cysts (23%), and Gartner’s duct cysts (11%) [2,3,7]. However, it is essential to exclude conditions with similar symptoms before diagnosing a vaginal cyst. These conditions include Skene’s duct cyst, ectopic ureterocele, urethral diverticulum, cystocele, and other pelvic organ prolapses.

To diagnose a vaginal cyst, ultrasound or MRI may be used. In this particular case, we relied on ultrasound, which helped determine the cyst location, size, and its relationship to nearby structures, and also assisted in ruling out the initial misdiagnosis of protruding fetal membranes. However, MRI is often the imaging modality of choice due to its advantages in visualizing the vagina and surrounding areas. It also offers multiplanar capabilities and high-contrast resolution. In our patient’s case, we did not use MRI because the ultrasound provided sufficient information. Moreover, histopathology analysis remains the only method for a confirmatory diagnosis [1,2]. Müllerian cysts are primarily lined by columnar endocervical-like cells, but may also be lined by endometrial or fallopian-type epithelium [8].

The management of vaginal cysts depends on their size and clinical presentation. Cysts that are under 4 cm and asymptomatic typically require no treatment and can be monitored with follow-up appointments. Conversely, cysts larger than 4 cm often present with bothering symptoms necessitating surgical intervention [2]. In our patient’s case, upon confirming the diagnosis of a vaginal cyst, we proceeded with cyst aspiration to temporarily relieve symptoms and facilitate the projected vaginal delivery. Regrettably, the patient’s attempt at vaginal delivery was unsuccessful, culminating in the termination of her pregnancy through an emergency cesarean section. This procedure was followed by vaginal cyst excision and repair of the vaginal wall as a definitive treatment. Besides cyst aspiration and excision performed on our patient, other surgical methods to manage vaginal cysts encompass marsupialization, unroofing, and puncture. Nevertheless, to prevent recurrence, complete cyst wall excision is considered the most effective approach. In instances where cyst remnants are unresectable, vaporizing the interior capsule may help to lower the recurrence rate [1,2].

## Conclusions

Large vaginal cysts during pregnancy are viewed as an atypical presentation, particularly when compounded with cervical incompetence, as demonstrated in this case. This presents a noteworthy diagnostic challenge. By increasing clinical awareness about these rare cases, along with adopting a targeted approach to patient history, performing a thorough physical examination, and appropriately utilizing imaging, it becomes feasible to facilitate early and accurate diagnosis.

Fortunately, our patient recovered without complications. We counselled her about breastfeeding and contraceptive use, and educated her about the possible remnant of the vaginal cyst, which could lead to recurrence. We also underscored the importance of early follow-up in future pregnancies. As for her regular vaso-occlusive crises due to sickle cell disease, a medical team consultation recommended starting hydroxyurea treatment postnatal, which she agreed to. We discharged the patient to her home in stable condition and scheduled a follow-up appointment at the obstetrics and gynecology outpatient clinic.
